# Plant resistance to tomato yellow leaf curl virus is enhanced by *Bacillus amyloliquefaciens* Ba13 through modulation of RNA interference

**DOI:** 10.3389/fmicb.2023.1251698

**Published:** 2023-10-06

**Authors:** Qiao Guo, Yifan Sun, Chenglong Ji, Zirong Kong, Zhe Liu, Yulong Li, Yunzhou Li, Hangxian Lai

**Affiliations:** ^1^College of Natural Resources and Environment, Northwest A&F University, Xianyang, China; ^2^College of Agriculture, Guizhou University, Guiyang, China

**Keywords:** *Bacillus amyloliquefaciens*, RNA interference, ARGONAUTE family, plant viral disease, antiviral defense

## Abstract

**Introduction:**

Tomato yellow leaf curl virus (TYLCV), which is a typical member of the genus *Begomovirus*, causes severe crop yield losses worldwide. RNA interference (RNAi) is an important antiviral defense mechanism in plants, but whether plant beneficial microbes used as biocontrol agents would modulate RNAi in defense against TYLCV remains unclear.

**Methods:**

Here, we employed whole-transcriptome, bisulfite, and small RNA sequencing to decipher the possible role of *Bacillus amyloliquefaciens* Ba13 as a bacterial biocontrol agent against TYLCV in RNAi modulation.

**Results:**

Potted tomato plants were exposed to whiteflies for natural viral infection 14 days after bacterial inoculation. Compared with non-inoculated controls, the abundance of TYLCV gene in the leaves of inoculated plants decreased by 70.1% at 28 days post-infection, which mirrored the pattern observed for plant disease index. The expression of the ARGONAUTE family genes (e.g., *AGO3, AGO4, AGO5*, and *AGO7*) involved in antiviral defense markedly increased by 2.44–6.73-fold following bacterial inoculation. The methylation level at CpG site 228 (in the open reading frame region of the RNA interference suppressing gene AV2) and site 461 (in the open reading frame regions of *AV1* and *AV2*) was 183.1 and 63.0% higher in inoculated plants than in non-inoculated controls, respectively. The abundances of 10 small interfering RNAs matched to the TYLCV genome were all reduced in inoculated plants, accompanied by enhancement of photosystem and auxin response pathways.

**Discussion:**

The results indicate that the application of *Ba. amyloliquefaciens* Ba13 enhances plant resistance to TYLCV through RNAi modulation by upregulating RNAi-related gene expression and enhancing viral genome methylation.

## Introduction

Tomato yellow leaf curl virus (TYLCV) is a single-stranded circular DNA virus of the genus *Begomovirus* (family Geminiviridae). Since it was first discovered in Israel, TYLCV has spread to many other places, including the Middle East, Mediterranean coast, Africa, and Asia (Lu et al., 2023). TYLCV is one of the most detrimental viruses to tomato cultivation and it causes severe crop yield losses in tropical and subtropical regions. Because TYLCV is transmitted through whiteflies (*Bemisia tabaci*) (Gafni, 2003), chemical pesticides are often used to control this virus disease (Palumbo et al., 2001). However, the long-term use of pesticides is harmful to the environment and easily induces resistance in *B. tabaci* (Cahill et al., 1996). Given its advantages such as low cost and environmental friendliness, the use of beneficial microbes as biocontrol strains has attracted increasing interest in the control of TYLCV disease.

Members of *Bacillus* (Kumar et al., 2016), *Pseudomonas* (Shen et al., 2014), and *Trichoderma* (Elsharkawy et al., 2013; Vitti et al., 2015) have been used to control plant diseases by improving plant systemic resistance. There are two main pathways of systemic resistance in plants (Wang et al., 2009). Systemic acquired resistance (SAR) is dependent on salicylic acid and the regulatory protein NPR1 (Mou et al., 2003), whereas induced systemic resistance (ISR) is modulated by hormones such as jasmonic acid and ethylene. It has been shown that plant beneficial microbes can activate disease resistance pathways by regulating the expression of resistance-related genes (Elsharkawy et al., 2012) and the biosynthesis of hormones (Niu et al., 2011), ultimately enhancing plant resistance to the virus.

In addition to systemic resistance, RNA interference (RNAi) is recognized as an important defense mechanism in plants against viral infection (Kanakala and Ghanim, 2016). RNAi can be divided into small interfering RNA (siRNA)-mediated post-transcriptional gene silencing (PTGS) and transcriptional gene silencing (TGS), depending on the site and mechanism of action (Arif et al., 2013). PTGS occurs in the cytoplasm to precisely target and degrade mRNA transcripts of specific genes, which has been widely demonstrated to play an essential role in plant resistance against viral infection (El-Sappah et al., 2021). This process is mainly mediated by 21–24 nt virus-derived siRNA cut by Dicer-like (DCL) nucleases (Zheng et al., 2019). RNA-directed DNA methylation (RdDM)-mediated TGS is another important mechanism of plant defense against geminiviral infection, and it inhibits virus replication by increasing the methylation level of cytosine on the viral genome (Raja et al., 2008). Among them, methylation of CpG sites is crucial for plant defense against virus invasion, and viruses even evade plant defense by reducing the frequency of CpG sites in the genome (de Pradena et al., 2020; Loew et al., 2020).

The occurrence of PTGS and TGS involves the ARGONAUTE (AGO) family proteins, which are effectors of RNAi in plants (Li Z. C. et al., 2019). A growing number of AGO proteins have been identified to participate in the antiviral defense processes in plants. In particular, AGO4 is a major component of TGS that has been widely studied. In the classical RNA polymerase IV-RdDM pathway, the siRNA binds to AGO4 to form an AGO4-siRNA complex. This complex then binds to polymerase IV to take part in methylation of virus genomic sequences that are homologous to the siRNA under the action of domains-rearranged methyltransferase 2 (Matzke et al., 2015). Additionally, AGO3 has been reported to play a vital role in abscisic acid (ABA)-mediated antiviral defense (Alazem et al., 2014). Evidence suggests that both AGO3 and AGO7 play antiviral roles during TCV infection (Zheng et al., 2019). However, it is still unclear whether inoculation with biocontrol strains could modulate AGOs to enhance plant antiviral defense.

A previous study has demonstrated the robust effects of a bacterial strain, *Bacillus amyloliquefaciens* Ba13, for plant growth promotion and TYLCV disease control in tomato (Guo et al., 2019). How the presence of *Ba. amyloliquefaciens* Ba13 modulates the RNAi-based antiviral system in the host plant remains unclear. Here we verified the biocontrol effects of *Ba. amyloliquefaciens* Ba13 against TYLCV and explored the underlying mechanisms from the RNAi perspective. We analyzed the changes in RNAi-related gene expression in TYLCV-infected tomato leaves, as well as the methylation rate of TYLCV genome and the abundance of antiviral siRNAs in plant cells, after bacterial inoculation. The aim of this study was to decipher the possible role of *Ba. amyloliquefaciens* Ba13 in the antiviral RNAi system against TYLCV. The results could provide new evidence for microbial control of viral plant diseases.

## Materials and methods

### Experimental materials

The biocontrol strain, *Ba. amyloliquefaciens* Ba13 (SUB3558699; GenBank: MG846076), was isolated from a stratified old manured loessial soil under a corn-wheat rotation system in Yangling, Shaanxi Province, China. The strain was preserved by the Resource and Environmental Biology Laboratory in the College of Resources & Environment, Northwest A&F University (Yangling, Shaanxi Province, China). Seeds of tomato (*Solanum lycopersicum* L.) cultivar “Jinpeng 1” were purchased from Xi’an Jinpeng Seedling Co., Ltd. (Xi’an, Shaanxi Province, China). To mimic natural viral infection, the TYLCV pathogen was sourced from viruliferous whiteflies (*B. tabaci*) collected in the field in Yangling.

### Seedling cultivation, bacterial inoculation, and viral infection

The pot experiment was carried out in a greenhouse on campus of Northwest A&F University. Plump tomato seeds were selected and surface sterilized with 75% ethanol for 30 s, followed by at least five washes with sterile distilled water. The sterilized seeds were placed evenly onto a filter paper in sterile Petri dishes (∼50 seeds per dish). To each dish, 2 mL of sterile water was added and the seeds were incubated in the dark at 26°C until germination. The germinated seeds were evenly sown into potted trays (50 holes per tray) containing seedling substrate and cultivated for 21 days. When true leaves formed, tomato seedlings with uniform growth were selected and transplanted into 60 pots with a diameter of 18.3 cm and a height of 12 cm (one seedling per pot). Each pot contained fertile field soil collected from a wheat-maize rotation system in Yangling. The soil was classified as Eum-Orthic Anthrosols (Staff, 2010). The soil was passed through a 2-mm sieve before use. The experimental used a randomized complete block design with three blocks, and each block contained 20 pots. The pots in each block were randomly and equally divided into two groups. One group was inoculated with *Ba. amyloliquefaciens* Ba13 (treatment group), while the other group was not inoculated (control group).

*Ba. amyloliquefaciens* Ba13 was cultured in beef extract–peptone broth at 37°C for 48 h with a shaking frequency of 180 rpm. The bacterial culture was diluted to 1 × 10^8^ colony-forming units (CFU) mL^–1^ with sterile phosphate-buffered saline. For the treatment group, 100 mL of diluted cell culture per pot was applied to the seedlings 7 days post-transplantation. The control group received 100 mL of sterile phosphate-buffered saline per pot. Natural viral infection was performed 14 days after bacterial inoculation. To do this, both non-inoculated controls and *Ba. amyloliquefaciens* Ba13-inoculated plants were placed in a greenhouse with whiteflies. Each block was covered with a 150-mesh high-quality nylon net of 1.2 m × 1.2 m × 1.5 m (length × width × height), and 1000 whiteflies were released under the net for infection over 4 weeks. The average temperature of the greenhouse was 28°C and the average light period per day was 12 h.

### Disease severity evaluation, growth measurement, and leaf sampling

The infection rate and disease severity of tomato plants in each group were determined at 14 and 28 days post-viral infection (dpvi). The disease severity was scored as follows (Sun et al., 2016): 0 = healthy plant without with curling yellow leaves; 1 = mild dwarfing (∼4/5 height of healthy plant) with curling yellow leaves (<20% on the top); 2 = dwarfing (∼2/3 height of healthy plant) with leaf curling and yellowing (<40% on the top); 3 = obvious dwarfing (∼1/2 to 2/3 height of healthy plant) with typical curling yellow leaves (60%); and 4 = severe dwarfing (<1/2 height of healthy plant) with typical curling yellow leaves (60% to all), or early withering. The disease index and control rate were calculated as follows.


Diseaseindex(%)

$$=\frac{\sum \left(\mathrm{Disease}\,\mathrm{severity}\times \mathrm{corresponding}\,\mathrm{plant}\,\mathrm{number}\right)}{4\times \mathrm{Total}\,\mathrm{number}\,\mathrm{of}\,\mathrm{plants}}\times 100$$

Controlrate(%)

$$=\frac{\begin{array}{c} \mathrm{Disease}\,\mathrm{index}\,\mathrm{of}\,\mathrm{control}\,\mathrm{group}\\ -\mathrm{disease}\,\mathrm{index}\,\mathrm{of}\,\mathrm{treatment}\,\mathrm{group} \end{array}}{\mathrm{Disease}\,\mathrm{index}\,\mathrm{of}\,\mathrm{control}\,\mathrm{group}}\times 100$$

Plant height was measured 28 dpvi and leaf samples were taken for subsequent analyses. Three biological samples (repetitions) were collected from each group, and each biological repetition was a mixture of three leaf samples collected from the same part of three random plants in each block. The sample consisting of the first and second leaves from the apical growing point was used for virus quantification; the third leaf was used for whole-transcriptome RNA sequencing (RNA-seq), bisulfite sequencing, and small RNA-seq, as well as for quantitative validation of functional genes; and the fourth leaf was used for the analysis of plant hormone contents. The leaf samples destined for virus quantification and sequencing analyses were rinsed with distilled water and blotted dry, then immediately wrapped with tin foil and placed in liquid nitrogen. The samples for hormone analysis were weighed and then placed into liquid nitrogen. All samples were brought to the laboratory where they were kept at −80°C until used.

### Virus genotyping and quantification

Total DNA was extracted from tomato plants using a modified hexadecyltrimethylammonium bromide (CTAB) method. PCR amplification was performed as described in the Supplementary Note 1, with previously reported primers for the full-length sequence of the complete TYLCV genome (Supplementary Table 1). The amplified products were sequenced to obtain the complete genome sequence. Then we determined the virus type by comparing the obtained sequence with available virus genotypes in the NCBI database. Gene sequence similarity analysis was performed using the MegAlign program in DNAstar (DNASTAR, Inc., Madison, WI, USA). The MEGA-X software (Kumar et al., 2018) and MUSCLE algorithm were used to align the nucleic acid sequences of multiple sequences, while the neighbor joining algorithm was applied to build phylogenetic trees for taxonomic analysis of the virus. There were 23 different TYLCV genotypes in the evolutionary tree (including the virus genotypes identified in this study). Their accession numbers in the NCBI database, origins, and the similarities to the genotype of the virus used in this study are listed in Supplementary Table 2. The search of virus open reading frame (ORF) was performed using the NCBI Open Reading Frame Finder.^1^

Quantification of TYLCV in tomato leaves was performed using the method of Sade et al. (2014). Total DNA was extracted from leaf samples using the M5 Plant Genomic DNA Kit (Mei5 Biotech Co., Ltd., Beijing, China). The concentration and purity of extracted DNA were measured using spectrophotometry with an ultra microplate (Epoch, BioTek, Winooski, VT, USA). The DNA integrity was detected using agarose gel electrophoresis. The DNA samples were subjected to real-time fluorescent quantitative PCR on an iQ5 PCR system (Bio-Rad, Hercules, CA, USA) as described in the Supplementary Note 1. The PCR amplification was performed using TYLCV-V1 as the primer (Supplementary Table 1) and the *EF1* gene as the internal reference. There were three technical replicates for each sample. Data collection and analysis were performed using the Bio-Rad iQ5 software (v2.1.97). The relative expression level of the viral gene was calculated using the 2^–ΔΔCT^ method (Schmittgen and Livak, 2008).

### Whole-transcriptome RNA-seq and gene functional analysis

Frozen leaf samples were sent to BGI Biotech Co., Ltd. (Shenzhen, China) for transcriptome sequencing using the BGISEQ-500 platform (BGI Biotech). Total RNA was isolated from the leaf samples using Trizol reagent (Invitrogen, Carlsbad, CA, USA) according to the manufacturer’s instructions. Then mRNA libraries were constructed. Whole-transcriptome RNA-seq data processing was performed as described in Supplementary Note 2. The detection of significantly differentially expressed genes (DEGs) was performed following the method described by Wang et al. (2010). Genes with a | fold-change| > 2 and *p*-value ≤ 0.001 were defined as DEGs.

The DEGs were subjected to Gene Ontology (GO) and pathway enrichment analysis using the PANTHER classification system^2^ (Mi et al., 2005) and the Kyoto Encyclopedia of Genes and Genomes (KEGG),^3^ respectively (Kanehisa et al., 2008). The basic local alignment search tool (BLASTn) was adopted to identify homologous genes in the tomato genome.^4^ The raw sequences were deposited at the Sequence Read Archive in the National Center for Biotechnology Information (NCBI)^5^ under BioProject accession number: PRJNA553064.

### Quantitative PCR validation

The validation of functional genes was performed by reverse transcription quantitative real-time PCR (RT-qPCR) using six genes involved in gene silencing, plant growth, and disease resistance. The PCR protocols are provided in Supplementary Note 3. There were three technical replicates for each sample, and the primer sequences used are provided in Supplementary Table 1. The relative expression levels of the selected genes and small RNAs were calculated as described for virus quantification.

### ABA analysis

The content of ABA in tomato leaves was measured using a previously reported method (Pan et al., 2010) as described in Supplementary Data (Note S4). Briefly, the ABA was extracted from leaf samples with 80% methanol/water (v/v) followed by a mixture of 5% acetic acid/ethyl acetate and water (1:1, v/v). The levels of ABA in the extracts were measured using gas chromatography coupled to triple quadrupole mass spectrometry (Agilent, Santa Clara, CA, USA).

### Viral genome methylation analysis by bisulfite sequencing

Gene-specific DNA methylation was assessed by a next generation sequencing-based BSP, according to previously published method (Gao et al., 2014). In brief, BSP primers were designed using the online MethPrimer software and listed in Supplementary Table 1. A total of 1 μg of genomic DNA was converted using the ZYMO EZ DNA Methylation-Gold Kit (Zymo Research, Irvine, CA, USA) and one twentieth of the elution products were used as templates for PCR amplification with 35 cycles using KAPA 2G Robust HotStart PCR Kit (Kapa Biosystems, Wilmington, MA, USA). For each sample, BSP products of multiple genes were pooled equally, 5′-phosphorylated, 3′-dA-tailed and ligated to barcoded adapter using T4 DNA ligase (NEB). Barcoded libraries from all samples were sequenced on Illumina platform. For the bisulfite sequencing reads of each sample, firstly, adapters and low-quality reads were removed using software Trimmomatic-0.36. After removing the adapter sequences and filtering out the low-quality reads, the clean sequencing reads were directly aligned to the target sequences using software Bsmap (v2.73) with the default parameters which combines genome hashing and bitwise masking to achieve fast and accurate bisulfite mapping. Methylation level of C base in TYLCV genome was calculated as follows.


MethylationrateofCsite(%)

$$=\frac{\mathrm{methylated}\,\mathrm{sequences}}{\mathrm{methylated}\,\mathrm{sequences}+\mathrm{demethylated}\,\mathrm{sequences}}\times 100$$

### Small RNA-seq and identification of virus-derived siRNA

Small RNA-seq was used to detected the changes in the types and abundances of siRNA in tomato leaves, which have lengths mainly concentrated in the range of 19–24 nt (Seo et al., 2013). For the sample preparation and library construction, small RNA-seq was performed using the BGISEQ-500 platform (BGI Biotech). Sample selection and total RNA extraction followed the same methods that were used for the whole-transcriptome RNA-seq. Small RNA libraries were then constructed and small RNA-seq data were processed as described in Supplementary Note 5. The siRNAs with a *p*-value ≤ 0.05 were considered significantly differentially expressed. The raw sequences were deposited at the Sequence Read Archive in the NCBI (see text footnote 5) under BioProject accession number: PRJNA553309.

Given that siRNAs can bind to viral mRNA for transcriptional and post-transcriptional inhibition of viral replication, we aligned all the differentially expressed (*p* < 0.05) siRNAs against the genome of the TYLCV-SJ which identified in this study. The alignment of sequences was performed using the SOAP2.22 short-read alignment software package (Li et al., 2009), with no mismatches allowed. For siRNAs that completely matched to the viral genome sequence, their name, sequence, matched site, abundance in the two treatment groups (*Ba. amyloliquefaciens* Ba13 and control), and corresponding viral genotype were recorded.

### Statistical analysis

Data were statistically analyzed using DPS 4.0.^6^ The comparison of group means was performed using the Student’s *t*-test, except that the disease index values obtained at two time points were compared using the least-significant difference (LSD) test. A *p*-value of less than 0.05 was considered significant. The relative expression levels (fold-changes) of genes and small RNAs were plotted using Excel 2010 (Microsoft Corp., Redmond, WA, USA), and the relative quantities (chromatographic peaks) of plant hormones were drawn using Origin 2022b (OriginLab Corp., Northampton, MA, USA). The enrichment of DEGs in the biotic stress pathway was analyzed using Mapman (Thimm et al., 2004).

## Results

### TYLCV genotype

Based on PCR amplification and sequencing analysis, the genotype of the virus present in the tomato leaves was identified to be TYLCV virion DNA TYLCV-SJ (GenBank accession number: MN910280.1). The PCR results (Supplementary Figure 1) showed that the total length of the viral genome was 2781 nt, which encoded eleven ORFs (>150 bp). The starting and ending sites and coding proteins of corresponding genes and their functions are listed in Table 1. According to the phylogenetic tree (Supplementary Figure 2) and the comparison of virus genotypes, TYLCV-SJ (MN910280.1) had the closest genetic relationship with TYLCV-JSYC (MF590740) and TYLCV-FJFZ (KX885028). The sequence of TYLCV-SJ was 98.1% similar to that of the Israeli virus genotype TYLCV-IS (X15656), and therefore was deemed to be a relative of the Israeli virus in China.

**TABLE 1:** Eight open reading frames corresponding to virus isolates used in this study.

	Gene	Position (nt)	Length (aa)	Encoded protein	Protein functions
Virus chain	*AV1*	308–1084	258	Coat protein	Unique known structural unit of virus particle; related to the packaging movement of virus; the main determinant of infection pathway of whitefly (Yaakov et al., 2011)
	*AV2*	148–498	116	AV2 protein	Participating in viral infection and suppressing RNA silencing (Wu et al., 2010)
	*AV3*	2350–2583	77	AV3 protein	Suppressing PTGS and TGS (Gong et al., 2021)
Complementary chain	*C1*	2615–1542	357	Virus replication-related protein	Initiating the rolling circle replication; its silencing impedes the growth and spread of TYLCV (Ramesh et al., 2007)
	*C2*	1633–1226	135	Transcription activator	Suppressing PTGS and TGS (van Wezel et al., 2002)
	*C3*	1485–1081	134	Reproduction enhancer	A replication accessory factor that enhances viral DNA accumulation (Sun et al., 2014)
	*C4*	2464–2171	97	C4 protein	Determining the disease phenotype (Padmanabhan et al., 2017)
	*C5*	862–655	67	C5 protein	Suppressing PTGS and TGS (Zhao et al., 2022)

### TYLCV disease control and plant growth promotion

**FIGURE 1:**
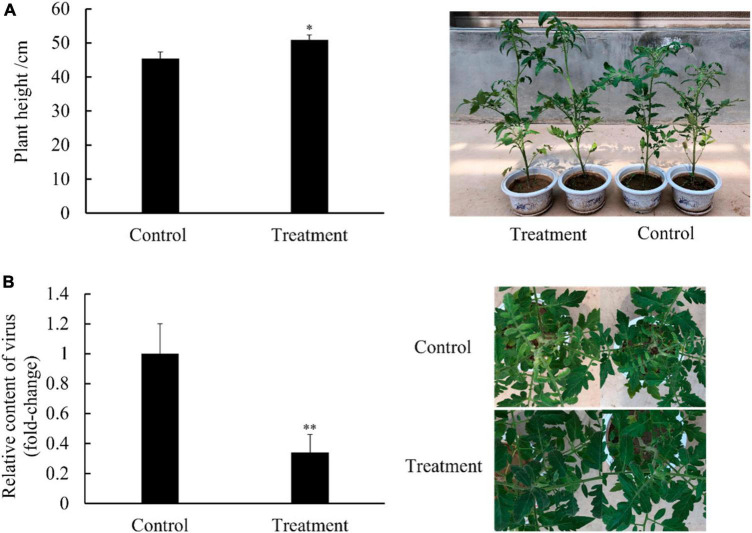
Effects of *Bacillus amyloliquefaciens* Ba13 in the promotion of tomato plant growth [**(A)**; *n* = 30, error bars = standard deviation] and defense against tomato yellow leaf curl virus disease [**(B)**; *n* = 3, error bars = standard deviation] at 28 days post-viral infection. **p < 0.01; *p < 0.05.

In the pot experiment, the tomato plants presented typical symptoms of TYLCV disease such as leaf curling and yellowing after viral infection. Compared with the control group, the plant height of the group treated with *Ba. amyloliquefaciens* Ba13 increased by 15.4% (*p* < 0.05; Figure 1A), while the relative abundance of the TYLCV gene in tomato leaves decreased by 70.1% at 28 dpvi (*p* < 0.01; Figure 1B). Additionally, the disease index of the treatment group was significantly lower than that of the control group at 28 dpvi (*p* < 0.05). The corresponding control rates in the treatment group were 38.7 and 32.1% at 14 and 28 dpvi, respectively (Table 2).

**TABLE 2:** The effect of *Bacillus amyloliquefaciens* Ba13 against tomato yellow leaf curl virus (TYLCV) disease.

Time (dpvi)	Group	Total number of plants	Disease index (%)	Control rate (%)
14	Control	30	21.7 ± 3.8bc	–
	Treatment	30	13.3 ± 5.2c	38.7
28	Control	30	41.7 ± 6.3a	–
	Treatment	30	28.3 ± 1.4b	32.1

dpvi: days post-viral inoculation. Disease index values are means of 30 replicates ± standard error. Different lowercase letters indicate significant differences between the control (TYLCV) and treatment (*Ba. amyloliquefaciens* Ba13+TYLCV) groups (Fisher’s LSD test; *p* < 0.05).

### Modulation of the leaf transcriptome

#### General data of the transcriptome

**FIGURE 2:**
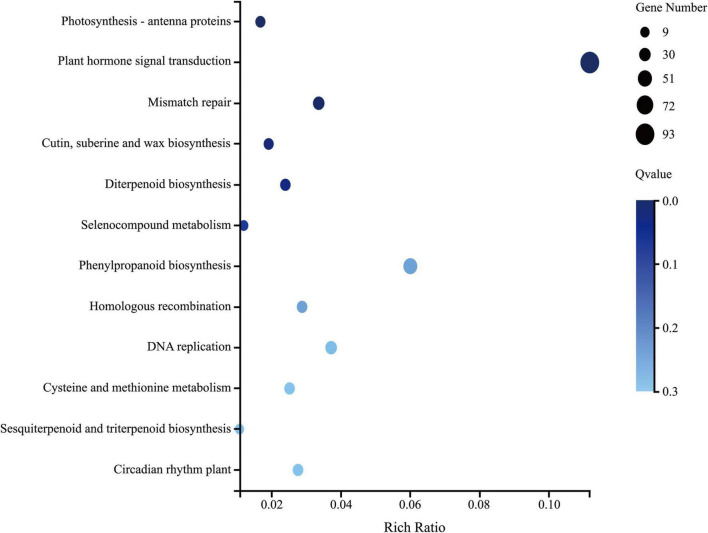
The 12 significantly enriched Kyoto Encyclopedia of Genes and Genomes (KEGG) pathways in tomato plants after treatment with *Bacillus amyloliquefaciens* Ba13.

Six leaf samples (three from each group) were analyzed using whole-transcriptome RNA-seq, which yielded 10.92 Gb of raw reads per sample. A total of 24,466 genes were detected, among which 1,897 genes (7.8%) were identified as DEGs with a | fold-change| > 2 and *p* < 0.001. There were 1352 DEGs upregulated and 545 DEGs downregulated in the group treated with *Ba. amyloliquefaciens* Ba13 relative to the control group. GO analysis showed that the DEGs were enriched in 2484 GO terms, 433 of which showed significant differences in expression. The 100 most significantly enriched GO terms, and the number of DEGs enriched in these terms, are provided in Supplementary Table 3. KEGG analysis revealed that a total of 126 pathways were enriched in the treatment group relative to the control group. Significant differences were found in 12 pathways including photosynthesis-antenna proteins, plant hormone signal transduction, and phenylpropanoid biosynthesis (*p* < 0.05; Figure 2).

#### Differential expression of functional genes

We specifically analyzed gene silencing-related genes after treatment with *Ba. amyloliquefaciens* Ba13. GO analysis showed that a total of eight DEGs including *AGO3*, *AGO4*, *AGO5*, and *AGO7* were enriched in the term gene silencing (GO: 0016458; Table 3). Multiple genes of the AGO family were upregulated in the RNAi system after treatment, with the fold-changes ranging from 2.03 to 6.73. The most pronounced upregulation was found in *AGO4*.

**TABLE 3:** Gene Ontology (GO) analysis of differentially expressed genes involved in gene silencing in tomato plants after treatment with *Bacillus amyloliquefaciens* Ba13.

Gene	Fold-change (Treatment vs. control)	*p*-value	Definition
*AGO3*	2.44	0.00029	ARGONAUTE RISC catalytic component 3
*AGO4*	6.73	1.90E-109	ARGONAUTE RISC component 4
*AGO5*	2.03	1.12E-105	ARGONAUTE family protein
*AGO7*	2.71	7.95E-69	ARGONAUTE family protein
*AGO10*	2.07	3.38E-128	Stabilizer of iron transporter SufD/Polynucleotide transferase
*LOC101261608*	6.89	9.99E-17	ARGONAUTE 4A-like protein
*RPA32a*	2.47	2.10E-187	Replication protein A
*LOC101255202*	2.72	5.29E-06	Protein INVOLVED IN DE NOVO 2-like

In addition to the silencing-related genes, a number of the identified DEGs were found to be involved in disease resistance pathways. *ABF*, a key gene involved in ABA biosynthesis, was upregulated 2.40-fold. Regarding salicylic acid biosynthesis, *NPR1*, an important defense signal regulatory gene downstream of salicylic acid was upregulated 4.58-fold. For mitogen-activated protein kinase (MAPK), eight DEGs related to serine/threonine-protein kinase OXI1 (OX1), mitogen-activated protein kinase kinase kinase (MAPKKK) ANP1, and mitogen-activated protein kinase kinase 4/5 (MKK4/5) were all upregulated between 2.06- and 4.72-fold.

Some DEGs were also found to be involved in growth regulation pathways. Regarding photosynthesis-antenna proteins, 14 DEGs involved in the biosynthesis of four light-harvesting complex II (LHCII) proteins including Lhca4 and Lhcb1 were upregulated between 2.03- and 3.07-fold (Supplementary Figure 3). For plant hormone signal transduction, there were 16 DEGs related to early auxin-responsive proteins including auxin-responsive protein/indoleacetic acid (AUX/IAA), small auxin-up RNA (SAUR), and Gretchen Hagen 3 (GH3). Of these 16 DEGs, 15 were upregulated between 2.01- and 236.14-fold. Additionally, three DEGs related to the auxin response factor (ARF) were upregulated, with a maximum fold-change of 3.81. Moreover, the physiological functions of brassinosteroids are similar to those of auxin; in relation to brassinosteroid biosynthesis, 12 DEGs participating in the biosynthesis of the proteins brassinosteroid insensitive 1 (BRI1), brassinosteroid resistant 1/2 (BZR1/2), and BRI1 kinase inhibitor 1 (BKI1), were upregulated. Supplementary Table 4 summarizes the details (name, involved pathway, and expression change) of the above-mentioned genes.

**FIGURE 3:**
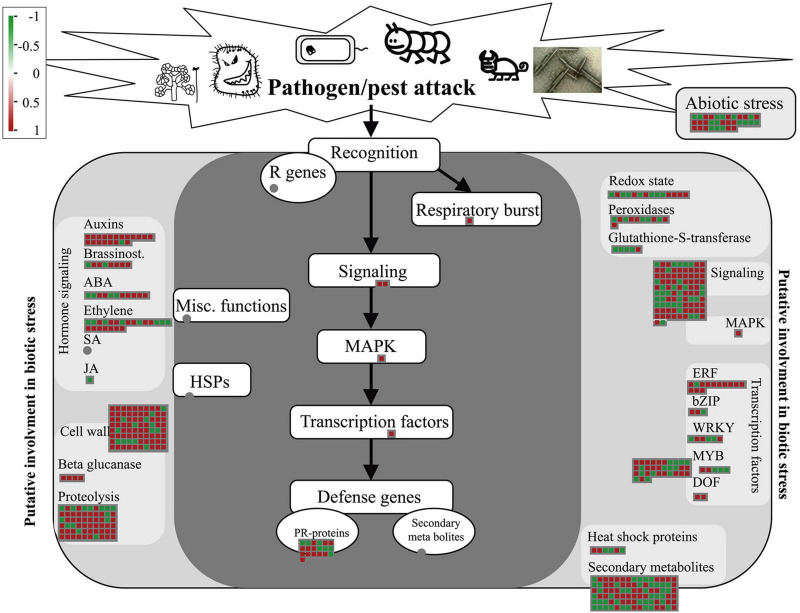
Gene expression changes of the biotic stress pathway in tomato plants after treatment with *Bacillus amyloliquefaciens* Ba13. Mapman was used to analyze the distribution of differentially expressed genes in the biotic stress pathway in the treatment group compared with the control group. Red squares indicate upregulated genes and green squares indicate downregulated genes.

The DEGs identified by whole-transcriptome RNA-seq were introduced into the corresponding biotic stress pathway in Mapman. Among the 1897 DEGs, 549 were enriched in this pathway. Following treatment with *Ba. amyloliquefaciens* Ba13, 97 of the 119 genes related to pathogenesis-related protein, auxin, and cell wall, were upregulated. Additionally, 67 of the 94 genes related to signaling and MAPK were upregulated (Figure 3). These results were consistent with the KEGG enrichment analysis of disease resistance-related genes.

#### PCR validation of functional gene expression

**FIGURE 4:**
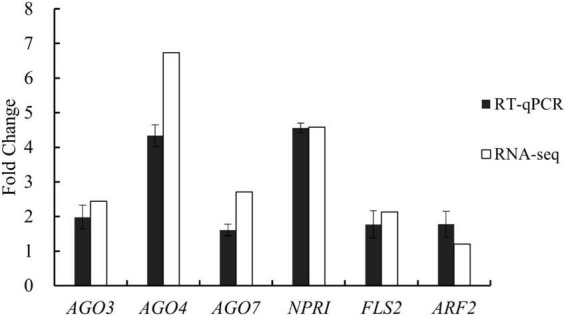
Expression changes of six genes involved in gene silencing, growth regulation, and disease resistance in tomato plants after treatment with *Bacillus amyloliquefaciens* Ba13 for validation of whole-transcriptome RNA sequencing (RNA-seq) data by reverse transcription quantitative real-time PCR (RT-qPCR).

The whole-transcriptome RNA-seq data were validated using RT-qPCR with six functional genes that were upregulated in the treatment group, with fold-changes of between 1.21 and 6.73. The PCR analysis revealed that all six genes increased their expression levels (between 1.61- and 4.34-fold) in the treatment group compared with the control group (Figure 4). The PCR results were generally consistent with the RNA-seq data. For example, according to the whole-transcriptome RNA-seq data, expression levels of *AGO4* (an important component of TGS), *FLS2* (a plant receptor kinase gene), and *NPR1* (a regulatory gene in the resistance system) increased 6. 73-, 2. 13-, and 4.58-fold, respectively; their expression changes were 4. 34-, 1. 77-, and 4.55-fold based on the RT-qPCR analysis. These results demonstrated the reliability of the whole-transcriptome RNA-seq data.

#### ABA content

**FIGURE 5:**
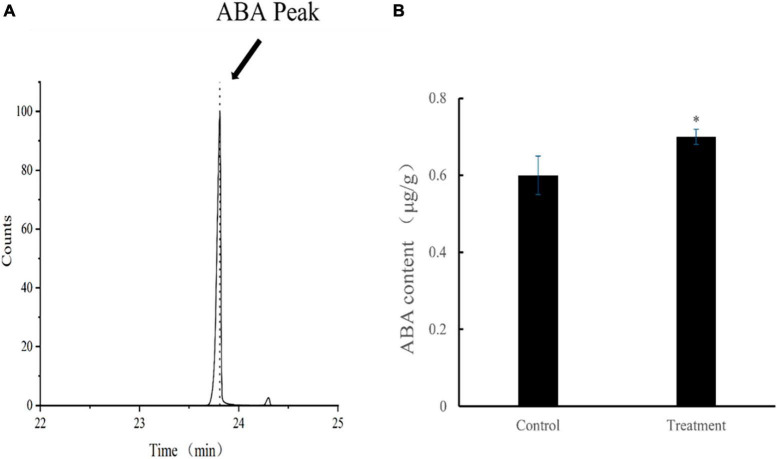
The content of abscisic acid (ABA) in leaves of tomato plants. **(A)** ABA standard retention time (23.787 min). **(B)** Changes in ABA content after *Bacillus amyloliquefaciens* Ba13 treatment compared with the control group. *n* = 3, error bar = standard deviation of ABA content in tomato leaves at 28 days post-viral infection. *p < 0.05.

The mean ABA content of tomato leaves in the treatment group was 693.3 ng/g at 28 dpvi, which was 15% higher compared with that of the control group (*p* < 0.05). The retention time and content of ABA after *Ba. amyloliquefaciens* Ba13 treatment are shown in Figure 5.

#### Modulation of viral genome methylation

**FIGURE 6:**
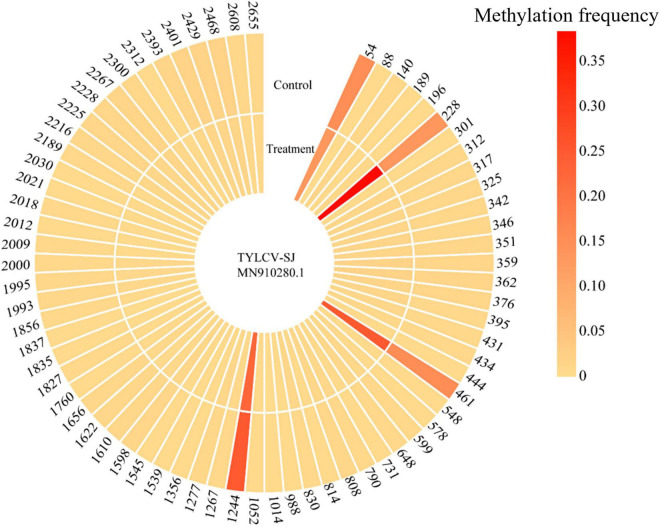
Changes in CpG methylation frequency of TYLCV genome after *Bacillus amyloliquefaciens* Ba13 treatment.

Bisulfite sequencing detected methylation on 69 of 81 CpG sites in the TYLCV-SJ genome. The methylation level of sites 54, 228, 461, and 1244 ranged from 13.3 to 38.4%, which was significantly higher than that of other CpG sites (0.3%–1.8%; Figure 6). Among them, site 228 was located in the ORF region of *AV1* and 461 sites were located in the ORF region of *AV1* and *AV2.* After *Ba. amyloliquefaciens* Ba13 inoculation, the methylation level of sites 228 and 461 was 183.1% and 63.0% higher than that of the control group, respectively (*p* < 0.01). However, the methylation level of sites 54 and 1244 did not significantly differ between the two groups.

#### Virus-derived siRNA abundances

In total, 15,448 siRNA sequences were obtained by small RNA-seq, 8480 of which had significant differences in their expression (*p* < 0.05). There were 4578 siRNA upregulated and 3902 siRNA downregulated in the treatment group compared with the control group. Ten siRNAs completely matched to the genome of the TYLCV genotype used in this study (MN910280.1), and their virus binding sites and sequences are provided in Table 4. All the 10 siRNAs were downregulated in the *Ba. amyloliquefaciens* Ba13 treatment group compared with the control group, with 21–22 nt possibly participating in RNAi. For example, siRNA5429 was matched to the ORF region of *AV1*, and siRNA5327, siRNA12690, siRNA16430, and siRNA15617 were matched to the ORF region of *C1*. Their abundances in the treatment group were reduced by 47.9, 39.7, 60.3, 99.7, and 62.7%, respectively.

**TABLE 4:** Changes in the abundance of 10 virus-derived small interfering RNAs (siRNAs) in the leaves of tomato plants after treatment with *Bacillus amyloliquefaciens* Ba13.

Name	Sequence (5′–3′)/Size	Log2 fold-change (Treatment vs. control)	*p*-value	Binding site (virus chain: +, complementary chain: −)	Corresponding gene
siRNA5327	CTCCATTTCTTTCTTCTTCTT/21 nt	−0.78	4.39E-5	−, 2077–2057	*C1*
siRNA5429	CGCATCTATTTCTATGATTCAA/22 nt	−0.98	4.97E-11	+, 1052–1073	*AV1*
siRNA12690	AAGCACTTCAAGGAATTCATG/21 nt	−1.43	1.99E-09	−, 1817–1797	*C1*
siRNA15142	AAATTCGGAAGTTGAGAAAA/20 nt	−5.65	2.52E-07	+, 1272–1291	–
siRNA15143	CAAATTCGGAAGTTGAGAAAA/21 nt	−4.39	0.0012	+, 1271–1291	–
siRNA15613	CAACACAAGATAGCCAAGA/19 nt	−0.64	7.49E-05	−, 1580–1562	*C1*, *C2*
siRNA15615	CCAACACAAGATAGCCAAGA/20 nt	−0.92	1.83E-14	−, 1579–1562	*C1*, *C2*
siRNA15617	TCCAACACAAGATAGCCAAGA/21 nt	−1.39	0.0068	−, 1561–1581	*C1*, *C2*
siRNA16430	ATTTAAAATATATGCCAAAAA/21 nt	−5.71	1.41E-07	−, 2604–2584	*C1*
siRNA16557	ATCTGGAACTTGATTAAAAGA/21 nt	−0.40	0.0094	+, 2040–2060	–

## Discussion

RNAi is an antiviral system which allow plants to directly act on viruses. The application of *Ba. amyloliquefaciens* Ba13 as a biocontrol strain against TYLCV necessitates a holistic understanding of how this strain modulates the RNAi-based antiviral system in its host plants. In the present study, we found that inoculation with *Ba. amyloliquefaciens* Ba13 increased the expression of RNAi-related genes (e.g., *AGO3*, *AGO4*, *AGO5*, *AGO7*) in tomato leaves, with improved levels of viral genome methylation and ABA content. These results indicate that the tomato plants might have initiated RNAi-related pathways after TYLCV invasion, whereas the application of *Ba. amyloliquefaciens* Ba13 could improve plant resistance by enhancing RNAi.

As an integral part of RNAi, DNA methylation-mediated TGS is considered to be an important pathway for plant defense against infection by geminiviruses (Mei et al., 2020). For DNA viruses, AGO4 is the main effector protein in the antiviral silencing machinery. Based on the whole-transcriptome RNA-seq data, we observed upregulation of *AGO4* expression in tomato leaves upon *Ba. amyloliquefaciens* Ba13 inoculation. Taking into account the role of AGO4–siRNA complex in viral genome methylation, we expected that *Ba. amyloliquefaciens* Ba13 mediated increased methylation level of the TYLCV genome. This hypothesis was supported by the higher methylation levels at CpG sites in *AV1* and *AV2* following *Ba. amyloliquefaciens* Ba13 inoculation. Previous study has found that after infection with the tomato leaf curl New Delhi virus, the methylation level of the viral intergenic spacer in a resistant cultivar was substantially higher than in a sensitive cultivar after infection. Additionally, Brough et al. (1992) introduced the cytosine methylated DNA of tomato golden mosaic virus into tobacco protoplasts, leading to a reduction in viral replication to 1/20–1/5 of the original level. These observations indicate that *Ba. amyloliquefaciens* Ba13 inoculation increased the methylation level of viral DNA through upregulation of *AGO4* expression, which in turn inhibited *AV1* and *AV2* expression and enhanced plant resistance to TYLCV. It has been shown that the methylation frequency of CpG sites in the plant genome markedly increases during virus invasion. CpG methylation frequency is a key factor in inhibiting geminivirus replication and reducing plant symptoms (Rodriguez-Negrete et al., 2013). Further study is needed to ascertain whether inoculation with *Ba. amyloliquefaciens* Ba13 can affect the frequency of plant genome methylation, thereby improving tomato resistance to TYLCV.

In addition to AGO4, other AGO family members (e.g., AGO3, AGO5, AGO7) play non-negligible roles in plant antiviral defense. AGO3 has been reported to take part in ABA-mediated antiviral defense processes (Alazem et al., 2014). Promoters of *AGO* genes contain several ABA responsive elements, and their expression levels are affected by exogenous ABA (Alazem et al., 2017). Therefore, ABA can enhance the expression of *AGO3*—which is necessary for plants to resist *Bamboo mosaic virus* (Alazem et al., 2017). In the case of TYLCV infection, *Ba. amyloliquefaciens* Ba13 inoculation increased *AGO3* expression in tomato plants. To clarify whether the upregulation of AGO3 expression is related to ABA, we looked at ABA biosynthesis related genes and ABA content in tomato leaves. We observed a remarkable upregulation in the expression of *ABF* upon *Ba. amyloliquefaciens* Ba13 inoculation, along with an increase in the ABA content. These results provide strong evidence that the application of *Ba. amyloliquefaciens* Ba13 improved ABA biosynthesis and enhanced the expression of AGO3, thereby facilitating plant defense against TYLCV.

With regard to *AGO5* and *AGO7* expression, we observed a distinct upregulation in tomato leaves upon *Ba. amyloliquefaciens* Ba13 inoculation. AGO5 can bind to small RNAs derived from viruses or viroids. Potato virus X (PVX) infection induces *AGO5* expression, which is essential for *Arabidopsis* to limit PVX infection (Brosseau and Moffett, 2015). AGO7 is also involved in PTGS and thus plays a role in plant defense against viruses (Garcia-Ruiz et al., 2015). For example, AGO7 has been shown to collaborate with AGO1 in clearing viral RNA with different levels of secondary structure (Qu et al., 2008). AGO7 additionally participates in plant defense against *Turnip crinkle virus*, and together with AGO3, it becomes the major contributor to virus clearance in the leaves (Zheng et al., 2019). The collective results allow us to conclude that the application of *Ba. amyloliquefaciens* Ba13 upregulated the expression of *AGO5* and *AGO7*, which synergistically acted to improve the efficiency of clearing viral RNAs in tomato plants.

While the expression levels of multiple *AGO* genes were upregulated in tomato plants inoculated with *Ba. amyloliquefaciens* Ba13, plant resistance to TYLCV was enhanced. It has been reported that an increase in *AGO* gene expression enhances plant resistance to different viruses such as *Bamboo mosaic virus*, Potato virus X, *Turnip crinkle virus* (Qu et al., 2008; Brosseau and Moffett, 2015; Alazem et al., 2017). This leads us to posit that *Ba. amyloliquefaciens* Ba13 is likely to regulate plant defense against other viruses by upregulating *AGO* expression. We additionally found that *Streptomyces pactum* Act12 can increase tomato resistance to TYLCV by upregulating *AGO* gene expression (Li Y. L. et al., 2019). This suggests that other biocontrol bacteria may perform similar functions as observed in *Ba. amyloliquefaciens* Ba13. More experiments are required to verify whether the effects and mechanisms of *Ba. amyloliquefaciens* Ba13 are universal for other viruses and biocontrol strains.

RNAi plays a role in disease control via siRNAs, which target viral mRNAs with corresponding sequences at specific sites for degradation. We compared the obtained siRNA sequences against the TYLCV genotype (MN910280.1) and found 10 siRNAs completely matched to our obtained viral sequence. The siRNA matcher site in the ORF were corresponding to *AV1*, *C1*, and *C2*. *AV1* and *C1* are, respectively, involved in coding virus coat protein and replication-related proteins, which help TYLCV growth and spread. *C2* can revert TGS and decrease DNA methylation in plants by interfering with the methyl cycle (Buchmann et al., 2009). The complete matching of these siRNAs to the functional gene regions of TYLCV indicates that the siRNAs may interfere with viral protein synthesis by complementary binding and degrading mRNA of gene transcription. This result illustrates that RNAi-related pathways were initiated in tomato plants in response to TYLCV infection.

We observed *Ba. amyloliquefaciens* Ba13-mediated downregulation of all 10 siRNAs matched to the TYLCV genotype in tomato leaves, consistent with the pattern observed in a previous study. Rodriguez-Negrete et al. (2009) investigated the recovery of plants (with less susceptible symptoms) after infection with pepper golden mosaic virus. They found a lower total abundance of virus-derived siRNAs but a higher DNA methylation level of the viral genome in the recovered tissue than in the diseased tissue of pepper plants. Additionally, we found that the disease symptoms of *Ba. amyloliquefaciens* Ba13-inoculated tomato plants were alleviated compared with those of non-inoculated controls. The decrease in siRNA abundances could be related to the decrease in virus quantity in tomato plants treated with *Ba. amyloliquefaciens* Ba13. *Ba. amyloliquefaciens* Ba13 inoculation enhanced plant defense against TYLCV, allowing faster recovery from viral infection.

Our results indicated that the expression levels of genes related to SA, ABA, and RNAi pathways were upregulated in tomato leaves after inoculation with *Ba. amyloliquefaciens* Ba13. In fact, the defense processes of plants against viruses involve the participation and collaboration of multiple mechanisms, such as RNAi, dominant resistance, and SAR (Nicaise, 2014). Thus, inoculation with *Ba. amyloliquefaciens* Ba13 is likely to induce multifaceted plant response through various defense systems. Although both SAR and RNAi play a role in plant defense against viruses, RNAi has higher specificity for viruses compared with the broad-spectrum resistance mechanism of SAR and can target degradation of viruses. While this study has focused on RNAi modulation by *Ba. amyloliquefaciens* Ba13, the relationship between RNAi and other defense mechanisms in inoculated plants should be clarified by further studies.

Furthermore, we found that the application of *Ba. amyloliquefaciens* Ba13 strongly affected the expression of genes related to photosystem and auxin response pathways. A total of 12 DEGs related to the biosynthesis of LHCII chlorophyll a/b binding protein 1 (LHCb1), LHCb3, and LHCb7 in the LHCII pathway (the most abundant light harvester in plants) were upregulated in tomato leaves upon *Ba. amyloliquefaciens* Ba13 inoculation. Additionally, 13 of the 14 DEGs related to auxin/indoleacetic acid and small auxin-up RNA, as well as two DEGs related to GH3, were upregulated in tomato leaves of inoculated plants. Montasser et al. (2012) found that the content of photosynthesis related chemical components (e.g., chlorophyll a, b) decreased in tomato leaves infected with TYLCV compared with normal leaves. Wu et al. (2012) showed that auxin/indoleacetic acid proteins were significantly expressed in tomato plants during root and stem development. Small auxin-up RNA plays a major role in modulating cell elongation and positively regulates cell expansion by modulating the transport of auxin (Chae et al., 2012). Furthermore, *GH*-like genes mediate the interaction between auxin and salicylic acid signaling pathways. Overexpression of *GH3* can enhance the systemic disease resistance in plants (Zhang et al., 2007). Taken together, these results indicate that the presence of *Ba. amyloliquefaciens* Ba13 could attenuate the negative effects of viral infection on leaf photosynthetic efficiency, promote plant growth, and as such, enhance plant defense against TYLCV.

## Data availability statement

The datasets presented in this study can be found in online repositories. The names of the repository/repositories and accession number(s) can be found below: https://www.ncbi.nlm.nih.gov/, MN910280.1, https://www.ncbi.nlm.nih.gov/, PRJNA553064, https://www.ncbi.nlm.nih.gov/, PRJNA553309.

## Author contributions

HL conceived the study and proposed the experimental approaches. QG, YS, and CJ conducted the experiments. QG, YS, ZK, ZL, and YZL contributed to data processing and analysis. QG, YS, and YLL wrote the manuscript. All authors read, commented on, and approved the final version of the manuscript.

## Footnotes

1
http://www.ncbi.nlm.nih.gov/gorf/gorf.html

2
http://www.pantherdb.org/data/

3
http://www.kegg.jp/

4
https://solgenomics.net/

5
https://www.ncbi.nlm.nih.gov/sra

6
http://www.dpsw.cn
