# Supplementary material for: Plant resistance to tomato yellow leaf curl virus is enhanced by Bacillus amyloliquefaciens Ba13 through modulation of RNA interference
Source: Front Microbiol. 2023 Oct 6;14:1251698. doi: 10.3389/fmicb.2023.1251698 (PMC10587425; doi:10.3389/fmicb.2023.1251698)
Supplement: Supplementary file 1 [file Data_Sheet_1.docx]

**Supplementary data for**

**Plant Resistance to Tomato Yellow Leaf Curl Virus is Enhanced by *Bacillus amyloliquefaciens* Ba13 Through Modulation of RNA Interference**

**Qiao Guo ^1†^, Yifan Sun^1†^, Chenglong Ji^1^, Zirong Kong^1^, Zhe Liu^1^, Yulong Li^1^, Yunzhou Li^2^, Hangxian Lai^1*^**

^1^ College of Natural Resources and Environment, Northwest A&F University, Yangling 712100, China, ^2^ College of Agriculture, Guizhou University, Guiyang 550025, China

*** Correspondence:**

Hangxian Lai

🖂 laihangxian@163.com

† These authors contributed equally to this work and share first authorship.

This paper contains 5730 words, with 4 tables and 6 figures.

**Note S1 PCR amplification for viral genotyping and virus quantification**

For viral genotyping, the 50 µL PCR reaction contained: 19 μL of double-distilled H_2_O, 25 μL of MiX, 2 μL of forward primer, 2 μL of reverse primer, and 2 μL of viral DNA template. The PCR program was as follows: pre-denaturation at 98°C for 3 min, followed by 35 cycles of denaturation at 98°C for 10 s, annealing at 56°C for 10 s, and extension at 72°C for 1 min.

For virus quantification, the 20 µL PCR reaction contained: 10 μL of NovoStart R SYBR qPCR SuperMix (SinoBio Biotech Co., Ltd., Beijing, China), 1 μL of DNA template, 1 µL each of forward and reverse primers (10 mM), and 7 µL of double-distilled H_2_O. The PCR program was as follows: pre-denaturation at 95°C for 5 min, followed by 40 cycles of denaturation at 95°C for 30 s, annealing at 55°C for 30 s, and extension at 72°C for 45 s.

**Note S2 Whole-transcriptome RNA-seq data processing**

Raw reads were first processed using SOAPnuke (parameters: -l 5 -q 0.5 -n 0.1) and then filtered using Trimmomatic (parameter: ILLUMINACLIP:2:30:10 LEADING:3 TRAILING:3 SLIDINGWINDOW:4:15 MINLEN:50). The obtained clean reads were aligned to the tomato reference genome using HISAT (Kim *et al.*, 2015) and then mapped to the reference sequence using Bowtie2 (Langmead and Salzberg, 2012). The gene and transcript expression levels of each sample were calculated using RSEM (Li and Dewey, 2011).

The open reading frame of each unigene was detected using getorf (http://emboss.sourceforge.net/apps/cvs/emboss/apps/getorf.html). The open reading frames were aligned to transcription factor domains from the PlnTFDB (Plant Transcription Factor database; http://plntfdb.bio.uni-potsdam.de/) using hmmsearch (http://hmmer.org). The function of unigenes was identified based on the characteristics of transcription factor families described by PlantTFDB (http://plntfdb.bio.uni-potsdam.de/v3.0/).

**Note S3 Quantitative PCR validation**

To validate the functional genes, the total RNA was rapidly extracted from tomato leaf samples using the RNA extraction kit (TransGen Biotech., Beijing, China). Reverse transcription was then performed using a reverse transcription kit (TransGen Biotech.) according to the manufacturer’s instructions. The total RNA (500 ng) was reverse transcribed into cDNA using random primers (Table S1). One microliter aliquots of the cDNA sample were used as the template for reverse transcription quantitative real-time PCR (RT-qPCR). The PCR reaction contained 10 μL of NovoStart R SYBR qPCR SuperMix (SinoBio Biotech., Beijing, China), 1 μL of cDNA template, and 1 μL of each of the forward and reverse primers (10 mM), with 7 μL of double-distilled H_2_O to make up a final volume of 20 µL. The PCR conditions were identical to those used for virus quantification.

**Note S4 Abscisic acid content analysis**

Fifty milligrams of each leaf sample were weighed into 2 mL EP tubes and ground with liquid nitrogen. The internal standard was added to each tube, followed by 1 mL of 80% (v/v) methanol/water. The tubes were kept at 4°C overnight and then centrifuged at 4900 *g.* The supernatants were collected and blown to dryness using nitrogen gas. The samples were mixed with 5% acetic acid/ethyl acetate and water (1:1, v/v) and centrifuged at 4900× *g*. The supernatants were collected and blown to dryness using nitrogen gas. Subsequently, each sample was mixed with 30 μL of methanol and 100 μL of water, and frozen at -20°C for an hour. After centrifugation (8800 *g*), the supernatants were collected into liner tubes and freeze-dried. N,O-Bis(trimethylsilyl)trifluoroacetamide (BSTFA) and pyridine (10:1, v/v) were added to each tube, and the reaction was allowed to proceed at 80°C for 30 min. The level of abscisic acid was measured using gas chromatography coupled to triple quadrupole mass spectrometry (Agilent, Santa Clara, USA).

**Note S5 Small RNA-seq data processing**

For the quantification of small RNAs, their expression levels were normalized using the transcripts per kilobase million (TPM) method (t Hoen *et al.*, 2008). The normalization was performed to eliminate the effect of different sequencing quantities on the accuracy of quantification. The normalized data were directly used for the subsequent comparison analysis. The TPM was calculated as follows:

$$TPM=(C\times10^6)/N$$

where *C* is the number of copies of a particular miRNA in the sample and *N* is the total number of reads mapped to the tomato genome in the sample.

Following their quantification, differential expression of the small RNAs was screened. RNA-seq is a random process, in which each sequence is uniformly and randomly derived from the respective sample (Jiang and Wong, 2009). Based on this hypothesis, the expression level of each gene (transcript) was considered to conform to a binomial distribution (or Poisson distribution). Using the above-mentioned model, the differential expression of small RNAs was calculated with DEGseq (Wang *et al.*, 2010) based on MA-plot (Yang *et al.*, 2002). Assuming C_1 and C_2 are the total number of aligned reads in samples 1 and 2, respectively, both of which conform to the binomial distribution, we define textit : {M} = log_2_ C_1 – log_2_ C_2, A= (log_2_ C_1 + log_2_ C_2)/2. It is proved that under the condition of random sampling, the distribution of M complies with A = a, which follows an approximate normal distribution.

**Table S1**

Primer sequences used in this study.

| Primer name | Forward primer (5'–3') | Reverse primer (5'–3') |
| --- | --- | --- |
| For virus quantification | | |
| *TYLCV-V1* | GGAAAAGCTTGCCTATGTGG | CCTGCAGCTTCCATACCAAT  (Sade et al., 2014) |
| For gene quantification | | |
| *EF1* | GACAGGCGTTCAGGTAAGG | CCAATGGAGGGTATTCAGC  (Wang et al., 2009) |
| *AGO3* | CTAGGCGGTCAAAATCCACC | GCCCATCGTTGAAGTGCCTT  (Kwon et al., 2020) |
| *AGO4* | TATTCTGCGGATTTGCCTTG | GGGTTTCTGTCTGGATTTCTC  (This study) |
| *AGO7* | TTAGCCCCTCCGTCGCTGCT | CCCCAACCATCGCGCTGAGA  (This study) |
| *ARF2* | CACGGAGGTTCAGGCAGATGCA | CAACGCAGGGGACAGCCGTC (Roodbarkelari et al., 2015) |
| *FLS2* | CTTGACAGTCCTAACGGT | TGTTATCTGCCACACTAAGAG  (Jeworutzki et al., 2010) |
| *NPR1* | GATGTGTGTGTTTGTGTGGACAACGAGT | CCATCGGATGTCAGATCAGAAGGTCTAG (Wang et al., 2009) |
| For high-throughput sequencing of TYLCV-SJ methylation levels | | |
| TY42-235 | GGATTTTATAGAYGTTATTGTTAATTAATTAAATTGTA | CCTCAACRAACTACAAATATTTAATAACTAACATACA |
| TY265-480 | GATTTAATTAGGGATCTTATATCTGTTGTAAGGG | CTATATATTCTAAACTTCCRATACATAAACCTATAC |
| TY510-681 | GATGTGAAGGCCCATGTAAAGTCCAATCTTATGAGCA | TCATCCATCCAAACTTTACCTAAAAAATATATC |
| TY691-865 | GAAGTAGAATTATATTAATTAGGTTATGTTTTTTTTGG | AACATAAAATTTCCTCATCACTTAAAACCTATCC |
| TY954-1141 | AGGAGGTAGTTAAGTATGAGAATTATATTGAA | TATACTTAAAAACACAATAAATATAACAACAACTCAT |
| TY1230-1394 | ATATTTTTAAGAAAYGATTAGTTTGAGGTTGTAATG | TAACAAAACACAACCAAAAACCATTTCTAATAAAC |
| TY1501-1709 | GAGGTAGTATGAGTAGTTATAGTTTAGGTTTA | CCAAAACCTACCTCCTCATATAAAAAATATCT |
| TY1722-1887 | GATAGTGGGAATTTTTTTTTTAATTTGAATGGGTT | CCTAAACCCAAAAATATACAATAATAATACATAATAC |
| TY1908-2069 | ATTATGTGGGTTTAGAGATTTGGTTTATATTGT | CTTTCTTCTTCTTTTAATCAAATTCCAAATAAACTTAAA |
| TY2150-2348 | AGTTTTTTGGGGTTTTTTTTTTTAATATATTGAGGGT | CAAACAACTAAAAACTCAACAAATATCAAAACCTAC |
| TY2361-2537 | GAAATGTGCTGACCTGTTTGGGGATACCAGG | CTTTCCCAATTAAAAAACCTAAAAACCCCAACAAAT |
| TY2557-2716 | GAATAATTGGGATATGTTAGGAAATAATTTTTGGTA | GTTTTACAAAATTACCAAATAACCATTAAATATCCA |

**Table S2**

Basic information of 23 virus genotypes used for phylogenetic tree construction.

| No. | Virus name | NCBI accession No. | Origin | Similarity with TYLCV-Yangling-SJ |
| --- | --- | --- | --- | --- |
| 1 | TYLCV-USA | EF539831 | California, USA | 99.2% |
| 2 | TYLCV-AU | KX347097 | Australia | 99.6% |
| 3 | TYLCV-Hawaii | GU322423 | Hawaii, USA | 99.6% |
| 4 | TYLCV-SC780 | MK559473 | China | 99.6% |
| 5 | TYLCV-Israel-AU | GU178816 | Queensland, Australia | 99.5% |
| 6 | TYLCV-Mexico | EF523478 | Mexico | 99.5% |
| 7 | TYLCV-JSYZ | MF590741 | Yangzhou, Jiangsu, China | 99.6% |
| 8 | TYLCV-Israel-CNSH | GU434144 | Shanghai China | 99.6% |
| 9 | TYLCV-BJ | KT338295 | Beijing China | 99.3% |
| 10 | TYLCV-SJ | MN910280 | Yangling, Shaanxi, China | 100% |
| 11 | TYLCV-JSYC | MF590740 | Yancheng, Jiangsu, China | 99.5% |
| 12 | TYLCV-FJFZ | KX885028 | Fuzhou, Fujian, China | 99.3% |
| 13 | TYLCV-XJ | JX456644 | Xinjiang, China | 99.4% |
| 14 | TYLCV-BJ2 | KT338293 | Beijing China | 99.2% |
| 15 | TYLCV-SX8 | JN412854 | Shanxi, China | 98.8% |
| 16 | TYLCV-SXYL2 | KC138545 | Yangling, Shaanxi, China | 98.8% |
| 17 | TYLCV-SXYL3 | KC138544 | Yangling, Shaanxi, China | 98.8% |
| 18 | TYLCV-SXYL4 | KC138543 | Yangling, Shaanxi, China | 98.9% |
| 19 | TYLCV-Netherlands | FJ439569 | Netherlands | 98.2% |
| 20 | TYLCV-IS | X15656 | Israel | 98.1% |
| 21 | TYLCV-Mild (Spain) | AF071228 | Spain | 90.4% |
| 22 | TYLCV | X63015 | Thailand | 51.4% |
| 23 | TYLCV-Iran | AJ132711 | Iran | 87.3% |

**Table S3**

The top 100 most significantly enriched Gene Ontology (GO) terms and the number of differentially expressed genes in each term of tomato plants after treatment with *Bacillus amyloliquefaciens* Ba13.

| ID | GO term | Candidate gene number |
| --- | --- | --- |
| GO:0005856 | Cytoskeleton | 36 |
| GO:0009768 | Photosynthesis, light harvesting in photosystem I | 13 |
| GO:0031409 | Pigment binding | 13 |
| GO:0000280 | Nuclear division | 28 |
| GO:0003677 | DNA binding | 200 |
| GO:0046906 | Tetrapyrrole binding | 70 |
| GO:1903046 | Meiotic cell cycle process | 25 |
| GO:0008574 | ATP-dependent microtubule motor activity, Plus-end-directed | 8 |
| GO:0006259 | DNA metabolic process | 66 |
| GO:0043596 | Nuclear replication fork | 9 |
| GO:0009765 | Photosynthesis, light harvesting | 14 |
| GO:0042555 | Minichromosome maintenance complex | 6 |
| GO:0008092 | Cytoskeletal protein binding | 36 |
| GO:0016168 | Chlorophyll binding | 14 |
| GO:0051321 | Meiotic cell cycle | 25 |
| GO:1990939 | ATP-dependent microtubule motor activity | 8 |
| GO:0140013 | Meiotic nuclear division | 23 |
| GO:0019915 | Lipid storage | 9 |
| GO:1903047 | Mitotic cell cycle process | 21 |
| GO:0051726 | Regulation of cell cycle | 29 |
| GO:0022414 | Reproductive process | 65 |
| GO:0018298 | Protein-chromophore linkage | 14 |
| GO:0000003 | Reproduction | 65 |
| GO:0048285 | Organelle fission | 30 |
| GO:0012511 | Monolayer-surrounded lipid storage body | 7 |
| GO:0000278 | Mitotic cell cycle | 21 |
| GO:0032300 | Mismatch repair complex | 16 |
| GO:0005874 | Microtubule | 18 |
| GO:0007346 | Regulation of mitotic cell cycle | 20 |
| GO:0030894 | Replisome | 6 |
| GO:0043601 | Nuclear replisome | 6 |
| GO:0005657 | Replication fork | 10 |
| GO:0051301 | Cell division | 21 |
| GO:1990391 | DNA repair complex | 16 |
| GO:0009522 | Photosystem I | 14 |
| GO:0010287 | Plastoglobule | 13 |
| GO:0009611 | Response to wounding | 16 |
| GO:0048037 | Cofactor binding | 117 |
| GO:0000075 | Cell cycle checkpoint | 8 |
| GO:0019748 | Secondary metabolic process | 39 |
| GO:0006310 | DNA recombination | 26 |
| GO:0048367 | Shoot system development | 28 |
| GO:0099080 | Supramolecular complex | 18 |
| GO:0099081 | Supramolecular polymer | 18 |
| GO:0099512 | Supramolecular fiber | 18 |
| GO:0099513 | Polymeric cytoskeletal fiber | 18 |
| GO:0005811 | Lipid droplet | 7 |
| GO:0051235 | Maintenance of location | 13 |
| GO:0004857 | Enzyme inhibitor activity | 29 |
| GO:0030983 | Mismatched DNA binding | 16 |
| GO:0005658 | Alpha DNA polymerase:primase complex | 4 |
| GO:0016491 | Oxidoreductase activity | 165 |
| GO:0007076 | Mitotic chromosome condensation | 4 |
| GO:0007131 | Reciprocal meiotic recombination | 17 |
| GO:0035825 | Homologous recombination | 17 |
| GO:1901987 | Regulation of cell cycle phase transition | 8 |
| GO:1901990 | Regulation of mitotic cell cycle phase transition | 8 |
| GO:0032502 | Developmental process | 90 |
| GO:0006298 | Mismatch repair | 16 |
| GO:0005976 | Polysaccharide metabolic process | 44 |
| GO:0060236 | Regulation of mitotic spindle organization | 6 |
| GO:0070507 | Regulation of microtubule cytoskeleton organization | 6 |
| GO:0090224 | Regulation of spindle organization | 6 |
| GO:0020037 | Heme binding | 56 |
| GO:0005634 | Nucleus | 259 |
| GO:0007127 | Meiosis I | 17 |
| GO:0003002 | Regionalization | 11 |
| GO:0019684 | Photosynthesis, light reaction | 16 |
| GO:0009523 | Photosystem II | 14 |
| GO:0071554 | Cell wall organization or biogenesis | 57 |
| GO:0048046 | Apoplast | 23 |
| GO:0005506 | Iron ion binding | 49 |
| GO:0098813 | Nuclear chromosome segregation | 12 |
| GO:0005618 | Cell wall | 40 |
| GO:0030312 | External encapsulating structure | 40 |
| GO:0010410 | Hemicellulose metabolic process | 15 |

**Table S4**

Pathways enriched for differentially expressed genes and their expression changes in tomato plants after treatment with *Bacillus amyloliquefaciens* Ba13.

| Gene | Annotation | Fold-change |
| --- | --- | --- |
| Photosynthesis - antenna proteins (14 genes) | | |
| LHCA4 | Light-harvesting complex I chlorophyll a/b binding protein 4 | 2.08, 3.07 |
| LHCB1 | Light-harvesting complex II chlorophyll a/b binding protein 1 | 3.03, 2.82, 2.90, 2.33, 2.32, 2.31, 2.46, 2.74, 2.99, 2.27 |
| LHCB3 | Light-harvesting complex II chlorophyll a/b binding protein 3 | 2.81 |
| LHCB6 | Light-harvesting complex II chlorophyll a/b binding protein 6 | 2.03 |
| Plant hormone signal transduction (31genes) | | |
| AUX1 | Auxin influx carrier | 2.27, 2.46 |
| ARF | Auxin response factor | 2.32, 3.81, 2.34 |
| IAA | Auxin-responsive protein IAA | 3.40, 2.71, 0.45 |
| GH3 | Auxin responsive GH3 gene family | 2.41, 3.76 |
| SAUR | SAUR family protein | 2.62, 2.01, 13.78, 2.55, 2.10, 2.20, 2.16, 2.43, 7.87, 4.92, 236.14 |
| BRI1 | Protein brassinosteroid insensitive 1 | 2.32, 2.17, 2.29 |
| BKI1 | BRI1 kinase inhibitor 1 | 2.51 |
| JAR1 | Jasmonic acid-amino synthetase | 38.74, 5.36, 8.15 |
| JAZ | Jasmonate ZIM domain-containing protein | 0.15, 2.12 |
| NPR1 | Regulatory protein N22PR1 | 4.58 |
| MAPK signaling pathway (27 genes) | | |
| MKK4_5 | Mitogen-activated protein kinase kinase 4/5 | 3.51, 4.69, 2.16, 4.72, 2.62 |
| SHOC2， | Leucine-rich repeat protein SHOC2 | 166.02 |
| FLS2 | Threonine-protein kinase FLS2 | 14.86, 4.21, 2.13, 0.50, 2.14, 2.32, 2.19, 2.04, 0.50, 0.36, 2.60, 2.51, 2.17, 2.38, 2.13, 0.47, 2.40, 4.09, 10.82, 0.30, 3.59 |
| Selenocompound metabolism (6 genes) | | |
| MET | 5-Methyltetrahydropteroyltriglutamate-homocysteine methyltransferase | 2.09, 5.46, 2.75, 2.91, 4.85, 2.59 |
| Diterpenoid biosynthesis (20 genes) | | |
| E5.5.1.13 | Ent-copalyl diphosphate synthase | 0.49 |
| E4.2.3.19 | Ent-kaurene synthase | 3.04, 2.45 |
| KAO | Ent-kaurenoic acid hydroxylase | 101.84 |
| E1.14.11.15 | Gibberellin 3-beta-dioxygenase | 2.23, 5.31, 5.71, 0.05, 0.31, 0.45, 9.84, 2.38, 0.05 |
| E1.14.11.13 | Gibberellin 2-oxidase | 2.00, 2.76, 0.25, 0.29 |
| VPS13A_C | Vacuolar protein sorting-associated protein 13A/C | 3.27, 3.44 |
| MAN2C1 | Alpha-mannosidase | 11.47 |

**
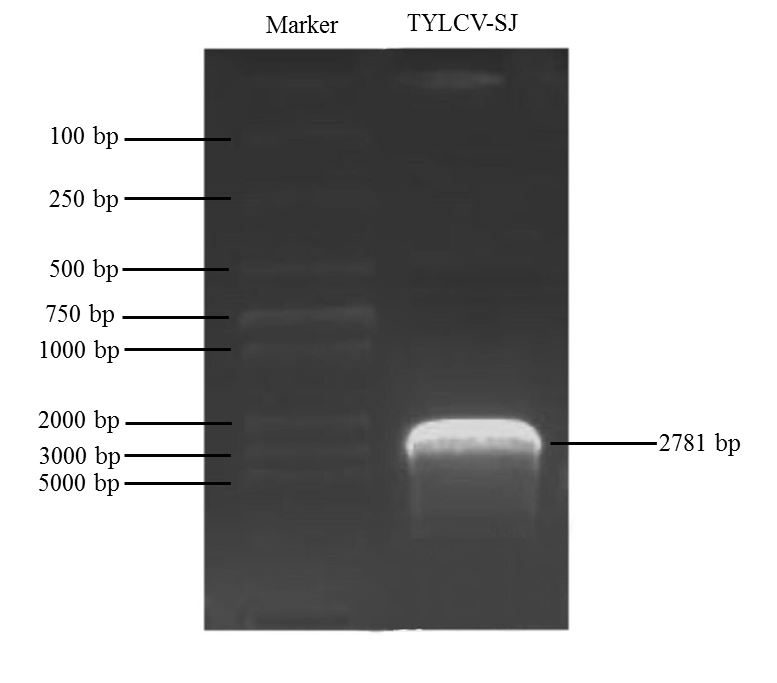
**

**Supplementary Figure 1.** Full-length PCR product of the viral genome.

**
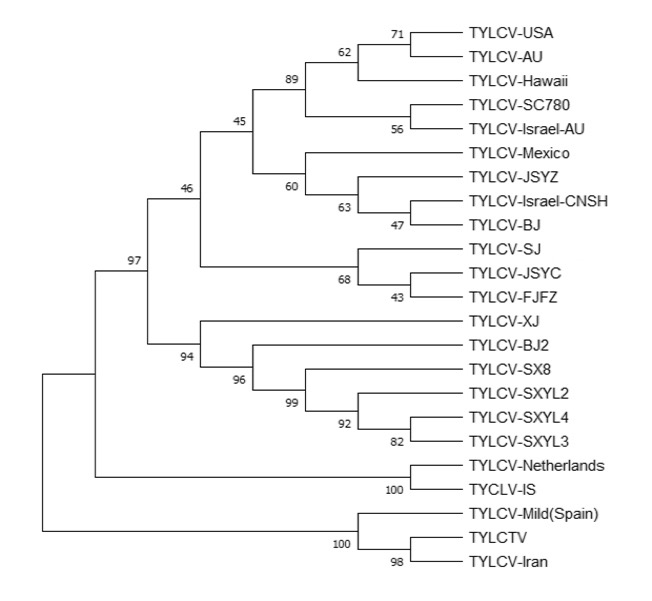
**

**Supplementary Figure 2.** Taxonomy of TYLCV-SJ.


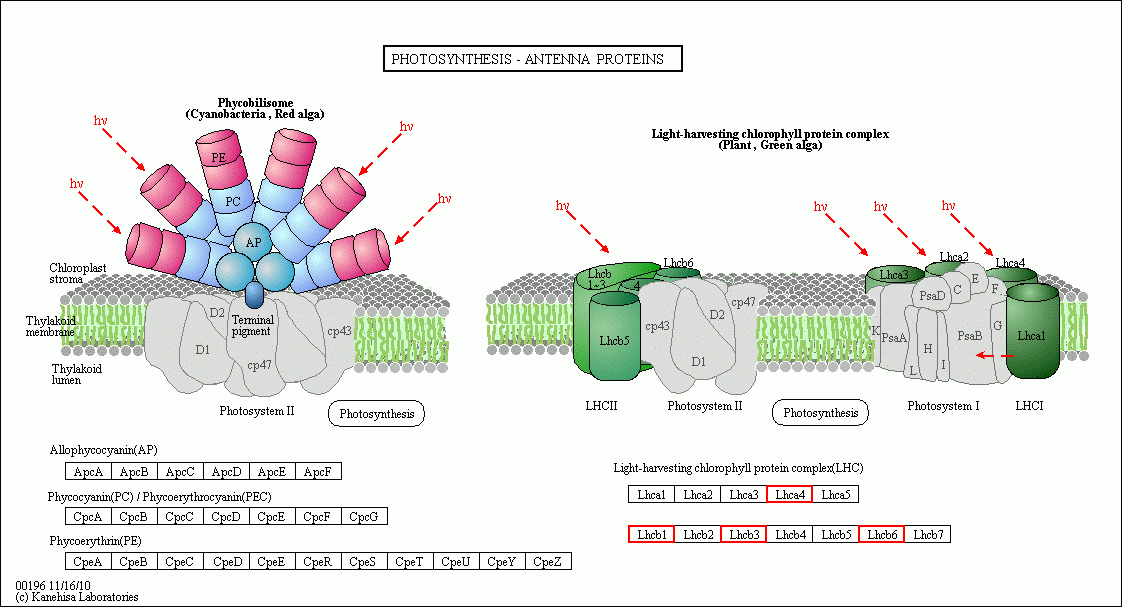


**Supplementary Figure 3.** Photosynthesis pathway in tomato regulated by the presence of *Bacillus amyloliquefaciens* Ba13. Red boxes represent upregulated genes, while blue boxes represent downregulated genes. Lhca4, Lhcb1, Lhcb3, and Lhcb6: light-harvesting complex II. Unigenes of these proteins are listed in Table S3.

**References**

Jeworutzki, E., Roelfsema, M.R.G., Anschütz, U., Krol, E., Elzenga, J.T.M., Felix, G., et al. (2010). Early signaling through the *Arabidopsis* pattern recognition receptors FLS2 and EFR involves Ca^2+-^associated opening of plasma membrane anion channels. *Plant J*. 62, 367–378. doi: 10.1111/j.1365-313X.2010.04155.x

Jiang, H., and Wong, W.H., (2009). Statistical inferences for isoform expression in RNA-seq. *Bioinformatics*, 25, 1026-1032. doi: 10.1093/bioinformatics/btp113

Kim, D., Langmead, B., and Salzberg, S.L., (2015). HISAT: a fast spliced aligner with low memory requirements. *Nat. Methods*, 12, 357-360. doi: 10.1038/NMETH.3317

Langmead, B., and Salzberg, S.L., (2012). Fast gapped-read alignment with Bowtie 2. *Nat. Methods*, 9, 357-359. doi: 10.1038/NMETH.1923

Li, B., and Dewey, C.N., (2011). RSEM: accurate transcript quantification from RNA-seq data with or without a reference genome. *Bmc Bioinformatics*, 12, 323. doi:10.1186/1471-2105-12-323

Roodbarkelari, F., Du, F., Truernit, E., and Laux, T., (2015). ZLL/AGO10 maintains shoot meristem stem cells during *Arabidopsis* embryogenesis by down-regulating ARF2-mediated auxin response. *BMC Biol.* 13, 74. doi: 10.1186/s12915-015-0180-y

Sade, D., Sade, N., Shriki, O., Lerner, S., Gebremedhin, A., Karavani, A., et al. (2014). Water balance, hormone homeostasis, and sugar signaling are all involved in tomato resistance to tomato yellow leaf curl virus. *Plant Physiol.* 165, 1684-1697. doi: 10.1104/pp.114.243402

t Hoen, P.A.C., Ariyurek, Y., Thygesen, H.H., Vreugdenhil, E., Vossen, R., de Menezes, R.X., et al. (2008). Deep sequencing-based expression analysis shows major advances in robustness, resolution and inter-lab portability over five microarray platforms. *Nucleic Acids Res*. 36, e141. doi:10.1093/nar/gkn705

Wang, L.K., Feng, Z.X., Wang, X., Wang, X.W., and Zhang, X.G., (2010). DEGseq: an R package for identifying differentially expressed genes from RNA-seq data. Bioinformatics 26, 136-138. doi:10.1093/bioinformatics/btp612

Wang, S.A., Wu, H.J., Qiao, J.Q., Ma, L.L., Liu, J., Xia, Y.F., et al. (2009). Molecular mechanism of plant growth promotion and induced systemic resistance to tobacco mosaic virus by *Bacillus* spp. *J. Microbiol. Biotechnol*. 19, 1250-1258. doi: 10.4014/jmb.0901.008

Yang, Y.H., Dudoit, S., Luu, P., Lin, D.M., Peng, V., Ngai, J., et al. (2002). Normalization for cDNA microarray data: a robust composite method addressing single and multiple slide systematic variation. *Nucleic Acids Res*. 30, e15. doi:10.1093/nar/30.4.e15
